# Changes in Critical Bronchiolitis After COVID-19 Lockdown

**DOI:** 10.7759/cureus.25064

**Published:** 2022-05-17

**Authors:** Jose Cardenas, Charlene Pringle, Stephanie L Filipp, Matthew J Gurka, Kathleen A Ryan, K. Leslie Avery

**Affiliations:** 1 Department of Pediatrics, Division of Critical Care Medicine, University of Florida, Gainesville, USA; 2 Department of Health Outcomes and Biomedical Informatics, University of Florida, Gainesville, USA; 3 Department of Pediatrics, Division of Infectious Diseases, University of Florida, Gainesville, USA

**Keywords:** viral coinfections, trend, severity of disease, respiratory syncytial virus (rsv), pediatric critical care, bronchiolitis, covid-19

## Abstract

Introduction

In response to the coronavirus disease 2019 (COVID-19) pandemic, state and local governments implemented mitigation strategies, including lockdowns, thereby averting the typical fall/winter 2020 bronchiolitis season and reducing the incidence of respiratory viruses, such as respiratory syncytial virus (RSV). Florida implemented a strict lockdown from April 1, 2020, to April 30, 2020. The removal of lockdown precautions on September 25, 2020, was followed by an atypical out-of-season surge of bronchiolitis in April 2021. Anecdotally, this surge appeared to be associated with both increased poly-viral coinfections and disease severity.

Objective

To determine if the bronchiolitis out-of-season surge differed from historical seasonal case patterns.

Methods

A single-center retrospective cohort study of admissions to the pediatric intensive care unit (PICU) with International Classification of Diseases, Tenth Revision (ICD-10) codes of bronchiolitis, from December 9, 2019, to February 29, 2020 (12 weeks, pre-lockdown group or PreLD), was compared to March 29, 2021, to June 19, 2021 (12 weeks, post-lockdown group or PostLD). Variables used for comparison were gender, ethnicity, age, viral coinfections, viruses detected, PICU length of stay, hospital length of stay, mortality, maximum respiratory support needed, mechanical ventilation days, extracorporeal life support (ECLS) days, and severity of disease measured by Pediatric Logistic Organ Dysfunction-2 (PELOD-2) and Pediatric Sequential Organ Failure Assessment (pSOFA). Categorical data were analyzed using Fisher's exact test, and a t-test was used for continuous variables. A two-sided p < 0.05 was considered significant.

Results

A total of 135 subjects were analyzed from the two cohorts. More patients were admitted during the PostLD phase (87 vs. 48). The PostLD group had a higher age at admission (11.2 ± 12.3 vs. 6.6 ± 7.5, p = 0.0075), but there were no differences in gender or race/ethnicity. The PostLD group also exhibited a higher proportion of RSV infections (73 vs. 16, p < 0.0001) and poly-viral infections (p < 0.0001). Higher coronavirus OC43 (9 vs. 0, p = 0.0263) and parainfluenza types 1-4 (human parainfluenza virus (HPIV)) (19 vs. 1, p = 0.0017) detections, yet fewer human metapneumovirus (HMPV) detections (0 vs. 4, p = 0.0147), were observed PostLD. No differences were found in hospital length of stay, PICU length of stay, mortality, mechanical ventilation days, ECLS days, or severity of illness scores based on PELOD-2 or pSOFA scores.

Conclusion

In the bronchiolitis out-of-season surge, there were an increased number of admissions to the PICU. Those patients were older, and more likely to have RSV, as well as a coinfection with coronavirus OC43 or HPIV, yet less likely to have HMPV. No difference in length of stay or disease severity was demonstrated.

## Introduction

In December 2019, a cluster of respiratory infections caused by a novel strain of coronavirus (SARS-CoV-2) linked to a seafood market in Wuhan, China marked the start of an unprecedented worldwide pandemic [1]. The coronavirus disease 2019 (COVID-19) pandemic was officially declared on March 11, 2020. To date, there have been over 270 million confirmed cases and more than 5 million deaths worldwide [2]. In response, many countries implemented lockdowns (e.g. permitting only essential activities to operate, initiating in-person school closures, and enforcing mandatory quarantine to travelers), social distancing, mandatory mask usage, and isolation of infected patients [3]. In the United States, most states implemented lockdown orders from late March to early April 2020, which were lifted beginning late April to early June 2020, with a varying degree of other precautions remaining in place depending on the state [4]. In the majority of counties in Florida, complete lockdown orders took place from April 1st through April 30, 2020, limiting only essential businesses to operate. By May 1, 2020, non-essential businesses operated with 25% capacity; by June 5, 2020, the allowed capacity increased to 50%, and personal care services were able to operate; by August 31, 2020, public schools reopened in person; and by September 25, 2020, all capacity restrictions were lifted [5].

The change in the implemented precautions led to a historically unusual and unexpected change in the incidence of respiratory infections. In the United States, respiratory viruses commonly follow a seasonal pattern from late fall through early spring. However, observations have demonstrated an initial decrease in respiratory syncytial virus (RSV) infections in the fall-winter months of 2020, followed by a significant increase in April 2021 [6]. Influenza viruses and human metapneumovirus (HMPV) infections remained low from March 2020 through May 2021, but increased infections by parainfluenza (types 1-4), adenovirus, and non-SARS-CoV-2 coronaviruses have been noted in the spring of 2021, followed by a decline in early 2020 [7], as well as an overall decreased incidence in invasive bacterial respiratory diseases in the winter of 2020 [8].

Bronchiolitis, which is most commonly caused by RSV, is the most common lower respiratory tract infection in children younger than two years of age. It is characterized by irritability, low-grade fever, nasal congestion, wheezing, and increased work of breathing and can cause severe respiratory symptoms, especially in children with underlying conditions [9]. Representing an important disease burden, bronchiolitis is the most common cause of hospitalization in the United States in children younger than two years of age. It comprises 18% of overall pediatric admissions and is the second most common cause of infectious deaths in infants in developing countries. Typically, seasonal outbreaks of bronchiolitis occur in the fall/winter months in the Northern Hemisphere including in the United States [10,11], with the exception of Florida having the longest outbreaks compared to the rest of the nation, with expected seasons from September through March in the northern region of the state [12].

Anecdotally, the pandemic-related out-of-season outbreak of bronchiolitis has been associated with multiple viral coinfections, increased severity of disease, more admissions to the pediatric intensive care unit (PICU), more interventions, and longer hospital stays. This study aimed to determine if this upsurge of cases from March through June 2021 did, in fact, have increased the severity of illness and medical support requirement compared with the typical seasonal spike of bronchiolitis from December 2019 through February 2020 at our institution. While the surge in cases post-lockdown has been described, no studies have yet been published examining the severity of illness comparisons.

## Materials and methods

We conducted, via chart review, a single-center retrospective cohort study of critically ill patients admitted to the PICU at UF Health Shands Children’s Hospital in Gainesville, Florida with a documented bronchiolitis diagnosis (International Classification of Diseases, Tenth Revision (ICD-10) codes: J21.9, J11.1, J21.8, J21.0, and J21.1), from December 1, 2019, through February 29, 2020 (pre-lockdown group or PreLD), and March 29, 2021, through June 19, 2021 (post-lockdown group or PostLD).

All data were entered and retained in the REDCap® database designed for this study. Demographic and viral indicators collected included patient sex, race/ethnicity, age at admission, RSV results, viral coinfections, and other viruses detected. Hospitalization and outcome metrics included PICU length of stay, hospital length of stay, and death before discharge. Illness severity was described by Pediatric Logistic Organ Dysfunction-2 (PELOD-2) [13] score at PICU admission, in addition to the Pediatric Sequential Organ Failure Assessment (pSOFA) [14] score for up to five sequential days following PICU admission, maximum respiratory support required, invasive mechanical ventilation days, and extracorporeal life support (ECLS) days.

Data management and analysis were conducted using SAS® 9.4 (SAS Institute Inc., Cary, NC). A predetermined level of α = 0.05 was chosen to evaluate statistical significance. Descriptive statistics were provided for all demographics and key hospitalization and illness severity metrics: frequencies and percentages for categorical data and mean ± SD for continuous measures. Comparisons were made between the "pre-lockdown" and "post-lockdown" time periods. Fisher’s exact tests and t-tests were used for comparison between the two periods for categorical and continuous outcomes, respectively. This study was approved by the Institutional Board Review of the University of Florida (RB202101398), and consent was waived.

## Results

A total of 135 patients were admitted to the PICU with an ICD-10 code of bronchiolitis during the two time frames analyzed. As seen in Table 1, almost twice as many patients with bronchiolitis were admitted during the PostLD phase compared with the usual winter spike of cases (PreLD). The PostLD group had a higher age of admission (11.2 ± 12.3 vs. 6.6 ± 7.5, p = 0.0075) but no difference in gender or race/ethnicity.

**Table 1:** Demographics of the 135 patients admitted with bronchiolitis to the PICU between the two time frames. Presented: categorical data: n (%); continuous data: mean ± SD. † Statistical testing: Fisher’s exact tests for categorical data; t-tests for continuous data. PICU: pediatric intensive care unit.

	Overall	Pre-lockdown	Post-lockdown	P-value
No. of patients	135	48	87	
Sex: male, n (%)	71 (52.6)	29 (60.4)	42 (48.3)	0.1763
Race/ethnicity				0.8202
Black, n (%)	37 (27.4)	13 (27.1)	24 (27.6)	
Hispanic, n (%)	17 (12.6)	5 (10.4)	12 (13.8)	
White, n (%)	73 (54.1)	28 (58.3)	45 (51.7)	
Unknown, n (%)	8 (5.9)	2 (4.2)	6 (6.9)	
Age at admission (in months, mean ± SD)	9.6 ± 11.0	6.6 ± 7.5	11.2 ± 12.3	0.0075

Microbiologically, the PostLD group had a higher proportion of RSV infections (83.9% vs. 33.3%, p < 0.0001). The distribution of RSV and the presence or absence of other coinfections also differed between PreLD and PostLD periods; a greater proportion had other infections without RSV during the PreLD period, and in contrast, during the PostLD period, a greater proportion had RSV with or without coinfection. In the PostLD period, there were a higher number of bronchiolitis cases caused by coronavirus OC43 (10.3% vs. 0.0%, p = 0.0263) and parainfluenza types 1-4 (21.8% vs. 2.1%, p = 0.0017), yet a lower proportion of HMPV (0.0% vs. 8.3%, p = 0.0147) (Table 2).

**Table 2:** Viruses detected in the 135 patients admitted with bronchiolitis to the PICU between the two time frames. Presented: categorical data: n (%); continuous data: mean ± SD. † Statistical testing: Fisher’s exact tests for categorical data; t-tests for continuous data. PICU: pediatric intensive care unit; RSV: respiratory syncytial virus.

	Overall	Pre-lockdown	Post-lockdown	P-value
No. of patients	135	48	87	
RSV+, n (%)	89 (65.9)	16 (33.3)	73 (83.9)	<0.0001
RSV and other viruses				<0.0001
RSV+/other+, n (%)	38 (28.2)	5 (10.4)	33 (37.9)	
RSV+/other-, n (%)	51 (37.8)	11 (22.9)	40 (46.0)	
RSV-/other+, n (%)	38 (28.2)	24 (50.0)	14 (16.1)	
RSV-/other-, n (%)	8 (5.9)	8 (16.7)	-	
Average count of other viruses detected, mean ± SD	0.7 ± 0.8	0.6 ± 0.5	0.8 ± 0.9	0.1963
Other viruses				
SARS-CoV-2/coronavirus, n (%)	2 (1.5)	-	2 (2.3)	0.5383
Adenovirus, n (%)	12 (8.9)	4 (4.2)	10 (11.5)	0.2119
Coronavirus 229E, n (%)	-	-	-	-
Coronavirus HKU1, n (%)	1 (0.7)	1 (2.1)	-	0.3556
Coronavirus NL63, n (%)	2 (1.5)	-	2 (2.3)	0.5383
Coronavirus OC43, n (%)	9 (6.7)	-	9 (10.3)	0.0263
Human metapneumovirus, n (%)	4 (3.0)	4 (8.3)	-	0.0147
Influenza A, *all subtypes, n (%)	1 (0.7)	1 (2.1)	-	0.3556
Influenza B, n (%)	1 (0.7)	1 (2.1)	-	0.3556
Parainfluenza, *all subtypes, n (%)	20 (14.8)	1 (2.1)	19 (21.8)	0.0017
Human rhinovirus/enterovirus, n (%)	46 (34.1)	20 (41.7)	26 (29.9)	0.1874

There were no differences between groups in hospital length of stay, PICU length of stay, death before discharge, mechanical ventilation days, ECLS days, or severity of illness scores based on the calculated PELOD-2 score and pSOFA (Table 3).

**Table 3:** Respiratory support requirement, length of stay, and measured severity of illness of the 135 patients admitted with bronchiolitis to the PICU between the two time frames. Presented: categorical data: n (%); continuous data: mean ± SD. † Statistical testing: Fisher’s exact tests for categorical data; t-tests for continuous data. PICU: pediatric intensive care unit; M.V: mechanical ventilation; ECLS: extracorporeal life support; CPAP: continuous positive airway pressure; BiPAP: bilevel positive airway pressure; pSOFA: Pediatric Sequential Organ Failure Assessment; PELOD-2: Pediatric Logistic Organ Dysfunction-2.

	Overall	Pre-lockdown	Post-lockdown	P-value
No. of patients	135	48	87	
Hospital length of stay (days, mean ± SD)	5.8 ± 9.2	5.4 ± 5.4	6.1 ± 10.8	0.6194
PICU length of stay (days, mean ± SD)	4.4 ± 7.8	3.9 ± 3.5	4.7 ± 9.3	0.4890
Deaths, n (%)	2 (1.5)	1 (2.1)	1 (1.2)	1.000
Maximum respiratory support (all)				0.2777
Low flow nasal cannula, n (%)	4 (3.0)	-	4 (4.6)	
High flow nasal cannula, n (%)	53 (39.3)	17 (35.4)	36 (41.4)	
CPAP, n (%)	9 (6.7)	5 (10.4)	4 (4.6)	
BiPAP, n (%)	46 (34.1)	20 (41.7)	26 (29.9)	
Mechanical ventilation, n (%)	21 (15.6)	6 (12.5)	15 (17.2)	
ECLS, n (%)	2 (1.5)	-	2 (2.3)	
Intubated patients (M.V. or ECLS), n (%)	23 (17.04)	6 (12.5)	17 (19.5)	0.3470
Mechanical ventilation only, n (%)	21 (15.6)	6 (12.5)	15 (17.2)	0.6211
pSOFA score, PICU day of admission (mean ± SD)	2.0 ± 2.2	2.0 ± 2.2	2.0 ± 2.2	0.8892
PELOD-2 score, PICU day of admission (mean ± SD)	1.3 ± 2.0	± 2.1	1.4 ± 1.9	0.2193
Mechanical ventilation days (mean ± SD)	6.7 ± 7.4	6.8 ± 5.1	6.7 ± 8.1	0.9719
ECLS days (n = 2), * individually reported	1.0 25.0	-	1.0 25.0	-
Patients with 5 days of pSOFA scoring (n)	31	12	19	
Among those with 5 days of pSOFA scoring: average scores (mean ± SD)	3.6 ± 3.4	3.3 ± 3.6	3.8 ± 3.3	0.7097

## Discussion

After easing precautions intended to mitigate the COVID-19 spread, a significant decline in the expected seasonal respiratory illnesses followed by an upsurge of out-of-season bronchiolitis cases has been reported in many parts of the world [6,7,12,15]. There has been speculation that cases during this upsurge were more severe, requiring more interventions and longer hospital stays. However, to our knowledge, no studies have closely examined the data between pre-lockdown and post-lockdown bronchiolitis patterns. Our study attempts to answer this uncertainty.

We found a larger number of admissions and higher age of admission in the PostLD group; this singularity can be explained potentially by the diminished exposure to common viruses during the winter months of 2020-2021, leading to an “immunity debt” [16] with no development of protective immunity, and in consequence, leaving a greater proportion of the population susceptible for infections like bronchiolitis at older ages [7,17]. This theory may also explain the larger proportion of patients with RSV detection and other co-viral infections, as well as the different sets of viral pathogens detected between groups, with higher detections of coronavirus OC43 and parainfluenza viruses and lower detections of HMPV in the PostLD group. It is noteworthy that viral co-infections with RSV are not known to be associated with increased illness severity, with the exception of HMPV, which is associated with increased risk of PICU admission and longer hospital stay [18], a detection that was absent during the PostLD period.

There was no significant difference in the hospital length of stay, PICU length of stay, or respiratory support requirement. However, in our cohort of patients, there was a slightly larger proportion of patients that required mechanical ventilation (17.2% vs. 12.5%) and ECLS (2.3% vs. 0%) support in the PostLD group. Though not statistically significant, further investigation with a larger sample size may be warranted.

No difference was found between groups in disease severity measured by PELOD-2 and pSOFA scores. It is worth noting that the PELOD-2 score is validated to detect multiorgan dysfunction syndrome (MODS) severity in the PICU [13]. However, in most cases, bronchiolitis presents with only respiratory symptoms and not MODS. Moreover, 87% of our subjects did not have arterial blood samples, thus potentially underestimating the respiratory dysfunction portion of the PELOD-2 score. The pSOFA score can potentially solve the problem of the lack of arterial blood samples since the oxygen saturation/fraction of inspired oxygen (FiO2) ratio can be used to measure oxygenation; however, hypoxemia is not always present in bronchiolitis. Frequently, patients with bronchiolitis require increased respiratory support based on their work of breathing and/or ventilation issues, which are parameters not captured by the pSOFA score component. Moreover, the pSOFA score is validated to discriminate pediatric in-hospital mortality [14], which is not optimal, considering the low mortality in our cohorts. Other validated scales for assessment of pediatric disease severity like the Pediatric Multiple Organ Dysfunction Score (P-MODS) [19] and the Pediatric Risk of Mortality III (PRISM III) [20] would have had the same limitations. A bedside clinical scale assessment tool such as the modified Wood’s Clinical Asthma Score (M-WCAS) [21] might have provided a better evaluation of the severity of illness in bronchiolitis; nevertheless, the retrospective design of the study made it impossible. Hence, demonstrating a difference in disease severity in bronchiolitis with a validated scale in our study was not feasible.

Our study is subject to several limitations. It was conducted at a single center with a small sample size, using a retrospective design, which limited available metrics. The disease severity might not have been well assessed by the scores used as described earlier, and the time frames analyzed in both cohorts do not include the entire bronchiolitis season. Complementary multi-centered studies are needed to further evaluate this unique out-of-season upsurge of bronchiolitis observed during the COVID-19 pandemic. A deeper understanding of the clinical and epidemiologic changes could provide useful information to guide future management of critical bronchiolitis cases, both during and after the current pandemic.

## Conclusions

In this single-center retrospective cohort study of critically ill patients admitted with bronchiolitis to the PICU, we found that in the PostLD out-of-season surge, there were an increased number of admissions with bronchiolitis to the PICU. Those subjects had a higher age of admission, more frequent RSV infections, more viral co-infections, a higher number of detections of coronavirus OC43 and all subtypes of parainfluenza, and less detections of HMPV. No difference in hospital length of stay or disease severity was demonstrated.
